# Some Remarks on Fumigations, and Baths of Caloric and Vapour, for the Consideration of Medical Practitioners

**Published:** 1825-09

**Authors:** J. Green

**Affiliations:** Member of the Royal College of Surgeons.


**Art. VI.**—*Some Remarks on Fumigations, and Baths of Caloric and Vapour, for the Consideration of Medical Practitioners.* By J. Green, Esq. Member of the Royal College of Surgeons.

The beneficial efficacy of the medicated gaseous, caloric, and vapour baths, may be depended on in so many morbid states of the body, that it would be taking up too much of the reader's time to enumerate them, as every practitioner will be enabled to draw his own inferences from the premises I am about to advance. I shall commence by stating the indications only, for which one or other of these baths is so essential as an auxiliary of the greatest importance, perhaps indispensable in the routine practice of the day; and, without giving cases in illustration of the subject, I trust it will at once appear to many of the profession as demanding their consideration. I do not hesitate to assert, from my practical knowledge of the effects of these baths, that more or less of the following indications are completely answered.

First indication.—Softening of the cutaneous texture, and relaxing parts affected by muscular or articular rigidity and phlegmasiæ.

Second.—Exciting the dermoid system, by producing perspiration and sweat.

Third.—Calming the nervous system, when exceedingly sensible.

Fourth.—Promoting the eruption of the exanthemata, and thus preventing metastasis.

Fifth.—The promotion of absorption in obstructions of the lymphatic glands, and the cellular membranes of the articulations.

Sixth.—Excitement of healthy action in the lost tone of the mucous membranes.

Seventh.—Restoration of the powers of muscular action, and of the digestive organs.

To attain any of these ends, the temperature should be from 110° to 130° of Fahrenheit, at which temperature it is pleasantly borne; and I venture to profess that, by the judicious use of such simple vapour or caloric baths, frequent or not, as the case may require, the expectations of both patient and practitioner will be fully realised. There are three indications which, I think, are met equally by the medicated fumigating baths, of sulphur, mercury, or chlorine:—

Eighth.—The facilitation or cure of most cutaneous diseases.

Ninth.—The cure of syphilis, or the sequel of syphilis.

Tenth.—The promotion of the cicatrisation of chronic ulcers.

When it is considered how many indications are thus answered, the reader is enabled to judge to what a variety of diseases this remedy is applicable, and it will be no longer a matter of surprise that extraordinary cures have been effected from this mode of practice, although directed by those possessing but crude conceptions of the rationale or principle by which it should be governed.

These remedies, however, should never be employed but with judgment, as their power over the animal economy is very great, and therefore capable of producing mischief. How imperiously, then, are medical men called on to take this practice more immediately under their own authority, and, by its judicious and wise application, add to their curative means an auxiliary which may be of so much utility; thereby preventing those evil consequences which must arise from its injudicious application.

Most persons are acquainted with the soothing effects of steam or vapour, and how effectually and immediately it cleanses the surface to which it is applied from all extraneous matter. Most persons, too, will recollect how imperfectly this object is attained by a warm-water bath, wherein both rubbing and soap are necessary to effect the same intention; for, without their assistance, the water runs off from the unctuous secretions and extraneous matter deposited on the surface of the skin. Vapour. to whatever applied at a sufficient temperature, occasions the papillæ of the skin to rise, and forces all the capillary vessels into increased action. Is it too much, then, to say, that the effects of simple vapour, at a sufficient temperature, is a powerful therapeutic agent, particularly when applied to the whole surface of the body? The excitement occasioned by the increased temperature quickens the pulse from forty to sixty beats in the minute; but, as the whole of the cutaneous surface is, as it were, breathing in a rarified atmosphere, the pulse, though so much quickened, is invariably soft, and feels large under the finger. After this state has occasioned the sweat to appear in drops on the forehead, which it does in ten or twelve minutes, it is proper for the patient to come out of the bath, or even before, if indicated by throbbing of the carotid or temporal arteries: otherwise, the excitement, if longer continued, may be dangerous, and, as I have said before, should not be indiscriminately used; yet it is too valuable a remedy in many cases to be overlooked.* With these precautions, who will deny that it is a remedy of great importance; for, in cases of superfluous internal heat and stimulus, what can be a better conductor of caloric than perspiration; and, when the body is in the opposite

* The injudicious mode of vapour-bathing, as introduced in this country, is objectionable, as the head is enveloped in the bath as well as the body, which can only be advisable in pulmonary cases, and at a low temperature.

state, what means are so likely, or more energetic than caloric, in reviving the suspended action of the organs, in sustaining and increasing the vital forces, and in re-establishing the equilibrium of the functions of the system? Who does not know the facility with which the absorbent cutaneous vessels imbibe, and carry into the circulation, the medicinal substances applied to the skin, when assisted by friction and heat? and, with these facts before us, what may not yet be effected by submitting the body to this increased temperature surrounded by the variously medicated gases, particularly if taken up by men of such acknowledged acumen and patient research as distinguish the medical men of this country?

The writer's view in this communication is to draw more closely the attention of medical men to this subject, under the conscientious belief that it merits their full consideration.

I shall conclude this paper by recording a case that lately came under my observation. A gentleman, in the spring of 1824, whilst on a visit at Romney, contracted tertian ague. This gentleman had received the best medical aid and attention, but with no advantage. In the spring of this year, 1825, he was directed to try my baths. He came nearly an hour before the attack of the usual paroxysm: his strength and appetite were gone, he was exceedingly emaciated, and his legs œdematous from debility. As this process was going to be tried, as it were, experimentally, I declined having him placed in the bath until some time after the cold trickling sensation down the back, and a distinct rigor, had come on. He was then placed in a simple caloric bath, with his face out: the cold sensations continued for ten minutes, his face assuming various hues, like the back of a marbled book-cover; it then gradually appeared with the usual flush that patients have when under the excitement of the bath, from which I judged the fit would be kept off. On coming out of the bath, he had no return of rigor. He had suffered so much from this fever, that he was dreadfully apprehensive of its return, and took five more baths before he left off, always coming a little before the time of the usual attack; but he never has had any relapse. All the medicine he took whilst using the baths was bark and port wine, in the same manner as he had long been in the habit of doing. I was surprised to see his rapid return of strength and appearance of health: long before he had taken the fifth bath, he had lost the œdema of the legs, and appeared perfectly convalescent.

*5, Bury-street, St. James's; May 11th,* 1825.

